# Changes of Mycobacterium tuberculosis specific antigen-stimulated CD27^−^CD38^+^IFN-γ^+^CD4^+^ T cells before and after anti-tuberculosis treatment

**DOI:** 10.1186/s40001-024-01713-x

**Published:** 2024-03-01

**Authors:** Yong Fang, Yuan Tang, Qiao-Xia Luo, Na Wang, Liang Tang, Xiao-Jun Yang, Xiao-Fang You, Yu-Chun Wang, Li Liang, Jing-Bo Zhang, Bo Su, Wei Sha

**Affiliations:** 1 Shanghai Clinical Research Center for infectious disease(tuberculosis), Shanghai Pulmonary Hospital, School of Medicine, Tongji University, Shanghai, 200433 People’s Republic of China; 2 Central Laboratory, Shanghai Pulmonary Hospital, School of Medicine, Tongji University, Shanghai, 200433 China; 3 The Third People’s Hospital, Tibet Autonomous Region, Lhasa, 850030 People’s Republic of China; 4 Department of Radiology, Shanghai Pulmonary Hospital, School of Medicine, Tongji University, Shanghai, 200433 People’s Republic of China; 5 Department of Occupational Disease, Shanghai Pulmonary Hospital, School of Medicine, Tongji University, Shanghai, 200433 People’s Republic of China

**Keywords:** Tuberculosis, Biomarkers, Flow cytometry, CD27, CD38, IFN-γ, Treatment monitoring

## Abstract

**Background:**

The aim of the study was to investigate whether the expression of CD27^−^CD38^+^ in interferon (IFN)-γ^+^CD4^+^ T cells stimulated by the specific antigen early secreted antigenic target-6 (ESAT-6)/culture filter protein-10 (CFP-10) could be a potential new therapeutic evaluation indicator for anti-tuberculosis (TB) treatment.

**Methods:**

Newly diagnosed active pulmonary TB patients, latent TB infection (LTBI) and healthy controls were enrolled from January 2021 to December 2021. PTB patients were treated by standard anti-TB regimen 2HREZ/4HR (2 months of isoniazid (H), rifampin (R), ethambutol (E), and pyrazinamide (Z) followed by 4 months of isoniazid (H) and rifampin (R)). The difference of CD27^−^CD38^+^ expression in IFN-γ^+^CD4^+^ T cells before treatment, 2 months after treatment, and 6 months after treatment were compared.

**Results:**

Total 45 PTB patients, 38 LTBI cases and 43 healthy controls were enrolled. The expression of CD27^−^CD38^+^ decreased significantly after anti-TB treatment and was comparable with that in LTBI and healthy controls when the 6-month anti-TB treatment course was completed. The decline rate of CD27^−^CD38^+^ between 6 months after treatment and baseline was positively correlated with erythrocyte sedimentation rate (*r* = 0.766, *P* < 0.0001), C-reactive protein (*r* = 0.560, *P* = 0.003) and chest computerized tomography severity score (*r* = 0.632, *P* = 0.0005). The area under receiver operator characteristic curve of CD27^−^CD38^+^ in distinguish pulmonary TB patients before and after treatment was 0.779.

**Conclusion:**

The expression of CD27^−^CD38^+^ in ESAT-6/CFP-10 stimulated IFN-γ^+^CD4^+^T cells can well reflect the changes of the disease before and after anti-TB treatment, which is expected to be a potential new therapeutic evaluation index.

*Clinical Registry number* chiCTR1800019966.

## Introduction

Tuberculosis (TB) is a contagious bacterial infection disease caused by *Mycobacterium tuberculosis* (MTB) [1, 2]. MTB most commonly infected lungs, named pulmonary TB (PTB) and communicable in this form. Aged adults, infants and people with weakened immune systems, are at higher risk of active TB or reactivation of TB. TB is one of the deadliest contagious diseases worldwide [2], especially in low-income countries, causing an estimated 1.1 million HIV-negative and 0.4 million HIV-positive people globally [3]. The standard regimen for treating TB is isoniazid (H), rifampin (R), ethambutol (E), and pyrazinamide (Z) (HREZ), which successfully treated 86%-95% patients [4]. However, PTB remains a major threat to global public health.

Early diagnosis and timely initiation of treatment is the key for blocking the transmission chain and preventing the emergence of drug-resistant cases, thus controlling the disease [5]. The major challenge for TB control is the monitoring of TB therapy and determining the success of TB treatment. The current diagnostic tests for PTB included sputum smear microscopy or sputum culture of acid-fast bacilli, Xpert MTB/rifampicin, chest radiograph, urine lipoarabinomannan and TB-loop-mediated isothermal amplification. Among these methods, sputum smear and sputum culture conversion are the commonly recommendations for TB treatment monitoring. However, these methods are less sensitive, time-consuming, and are easy to contaminate [6], reducing their usefulness as clinical treatment efficacy assessment tools. In addition, it is estimated that 20%-50% active PTB patients are smear-negative and sputum bacteriological testing are difficult to monitor the TB therapy. Therefore, development of alternative diagnostic methods is still worthy for investigation for improving TB control.

In recent years, study of different T cell subsets is an attractive option to monitor TB treatment. The frequencies of activated MTB-specific CD4^+^ interferon (IFN)-γ^+^ T cells are higher in active PTB patients than those in LTBI and healthy controls after being stimulated with early secreted antigenic target-6 (ESAT-6)/culture filter protein-10 (CFP-10) or purified protein derivative before anti-TB treatment initiation [7, 8]. Based on previous studies [9–14], MTB-specific CD4^+^ T cell activation markers of CD27, CD38, IFN-γ, HLA-DR and Ki-67 are able to distinguish active TB and LTBI, especially the expression of CD27^−^CD38^+^ in IFN-γ^+^CD4^+^ T cells can be used as an effective marker for the differential diagnosis of active PTB, latent tuberculosis infection (LTBI) and healthy controls. This study aimed to detect the expression of CD27 and CD38 in IFN-γ^+^CD4^+^ T cells in the peripheral blood of active PTB before and after treatment, and evaluate the value of their expression in evaluating the efficacy of active PTB treatment.

## Materials and methods

### Patients

This study has been registered in China Clinical Trials Registry (http://www.chictr.org.cn) with the registration number of chiCTR1800019966. This study complied with the Declaration of Helsinki and was approved by the Ethics Committee of Shanghai Pulmonary Hospital, School of Medicine, Tongji University, and all subjects signed an informed consent.

PTB patients, LTBI and healthy control were enrolled at Shanghai Pulmonary Hospital, Shanghai Pulmonary Hospital, School of Medicine, Tongji University from January 2021 to December 2021. The inclusion criteria of PTB patients were (1) newly diagnosed active PTB patients; (2) aged 18–70 years old. The diagnosis criteria of PTB included (1) no previous history of TB; (2) with typical clinical symptoms of TB, such as low-grade fever in the afternoon, night sweats, fatigue, and weight loss; (3) positive in interferon gamma release assay (IGRA) test; (4) positive bacteriological test for TB in sputum, bronchoscopy lavage fluid or lesion tissue, including positive in acid-fast staining and Bectec960 test, or positive in Gene-XPERT test; (5) the lesion site showed typical pathological manifestations of TB, including epithelioid cells, Langhans giant cells, and caseous necrosis; (6) chest CT examination support the diagnosis of PTB; (7) systemic and local anti-TB chemotherapy was effective and (8) other lung diseases were excluded. Among these criteria, the first and fourth ones must be satisfied.

The exclusion criteria were: (1) patients with primary or secondary immunodeficiency, including acquired immune deficiency syndrome (AIDS) patients, long-term use of hormones and patients with autoimmune diseases; (2) diabetic patients; (3) patients with viral hepatitis; (4) patients received anti-TB chemotherapy; (5) Bectec960 test suggesting non-tuberculous mycobacteria (NTM) infection; (6) Bectec960 test suggesting drug resistance.

In addition, LTBI participants and healthy individuals were enrolled as controls. The LTBI participants were enrolled from the close contacts of newly diagnosed TB patients who are IGRA positive and have no clinical evidence of active TB disease. The healthy controls were IGRA-negative healthy volunteers.

### Treatment

PTB patients received standard primary anti-TB regimen 2HREZ/4HR [9–12]. If drug-induced liver injury occurs during the treatment, the drug therapy was stopped and the treatment plan was adjusted.

The acid-fast stain and BECTEC MGIT 960 culture were performed on sputum before and every month after treatment. Computed tomography (CT), erythrocyte sedimentation rate (ESR), C-reactive protein (CRP) were examined before treatment, and at 2 and 6 months after treatment. Blood with heparin anticoagulant were extracted, stimulated with TB-specific antigen ESAT-6/CFP-10 for 16 h. The red blood cells were lysed, and then stained with fluorescent antibodies. The IFN-γ/CD4/CD38/CD27 expression were further analyzed using flow cytometry.

### CT scan

Lung high-resolution CT scanning were performed with Philips Brilliance iCT256 slice CT scanner (Philips, USA) with patients in a supine position. Scanning parameters were set as follows: tube voltage 120 kV, tube current 50–300 mA, collimator width 128 × 0.625 mm, screw pitch 0.758, matrix 512 × 512, slice thickness 1 mm, spacing 0.5 mm, and field of view 350 mm.

### Chest CT severity score

The severity score of lung lesions on chest CT was evaluated by two experienced radiologists from Shanghai Pulmonary Hospital following previous studies [13, 14] according to the distribution of lesions and the extent of involvement. The scores of the two physicians were added up to obtain the CT lung lesion severity score of a patient. Scores were performed before treatment, 2 months after treatment, and at the end of treatment.

### ESAT-6/CFP-10 stimulation

Whole blood with heparin anticoagulant was added with 10 μg/ml ESAT-6/CFP-10 polypeptide antigen within 4 h after collection. Golgi transport inhibitor brefeldin A (10 μg/ml, Biolegend, USA) was then added. After mixing, the sample was incubated for 16 h at 37 °C, 5% CO_2_. Subsequently, 2.5 ml of erythrocyte lysate and fixative (3% diethylene glycol, 2% formaldehyde and 0.75% methanol) was added and incubated at room temperature (25 °C) for 10 min. The cells were centrifuged at 600×*g* for 5 min, the supernatant was discarded, and 375 µl PerFix-nc (Beckman Coulter, USA) was added. IFN-γ-FITC, CD4-PE, CD3-ECD, CD27-PE-Cy5.5 and CD38-Pe-Cy7 were added and incubated in the dark for 45 min. Then, phosphate buffered saline (PBS) was added and centrifuged at 600×*g* for 5 min. The supernatant was discarded. The IFN-γ/CD4/CD38/CD27 expression were analyzed using flow cytometry (Beckman Coulter DxFLEX, Beckman Coulter, USA) and data were analyzed by using FlowJo V10 (BD Bioscience, San Jose, CA, USA).

### Statistical analysis

Statistical analysis was performed using GraphPad Prism 6 (GraphPad Software Inc., San Diego, CA, USA). Differences between paired baseline, and 2-month samples and 6-month samples were analyzed using a Wilcoxon matched-paired rank test or a Kruskal–Wallis when applicable. Receiver operator characteristic (ROC) curve analysis was used to test the ability of frequencies of CD27^−^, CD38^+^, and CD27^−^CD38^+^ distinguish treated and untreated PTB. A two-sided *P* < 0.05 was considered statistically significant.

## Results

### Characteristics of enrolled participants

**Fig. 1:**
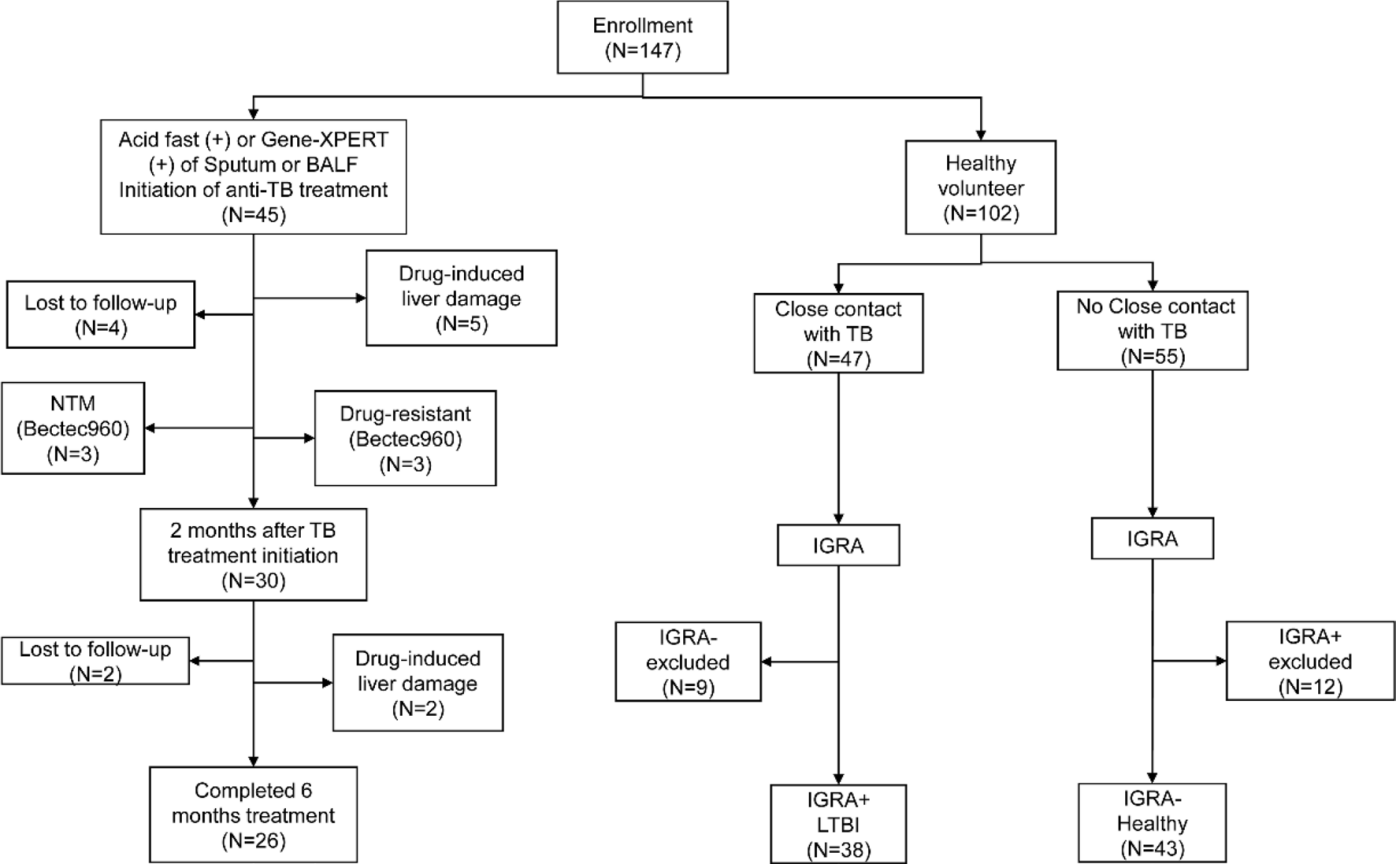
The flowchart of participant enrollment. *TB* tuberculosis, *BALF* bronchoalveolar lavage fluid, *LTBI* latent tuberculosis infection, *NTM* non-tuberculous mycobacterium pulmonary disease, *HC* healthy control, *IGRA* interferon-γ release assay

Total 45 PTB patients, 38 LTBI cases and 43 healthy controls were enrolled. Among the 45 PTB patients, 2 patients were NTM, and 2 patients have drug resistance. In the first 2 months of treatment, 5 cases demonstrated drug-induced liver damage and were discontinued from the treatment plan, and 4 cases were lost to follow-up. Between the 2 and 6 months of treatment, 2 cases of liver damage occurred and 2 cases were lost to follow-up. Finally, 26 subjects completed the 6-month course of treatment and follow-up. The flowchart is shown in Fig. 1. The baseline characteristics of the recruited PTB patients, LTBI participants, and healthy individuals are shown in Table 1. Twenty-one PTB patients became negative in BECTEC MGIT 960 culture of sputum at 1 month after treatment. Two cases became negative at 2 months after treatment, and 3 cases turned negative at 3 months after treatment. The chest CT lung severity score of the 26 patients were significantly reduced from 20.92 ± 6.88 before treatment to 15.27 ± 7.08 at 2 months after treatment and 10.54 ± 7.43 after 6 months of treatment.

**Table 1:** Characteristics of the participants

	PTB (*n* = 26)	LTBI (*n* = 38)	HC (*n* = 43)	P*
Male (*n*, %)	16 (61.5%)	15 (39.5%)	16 (37.2%)	*P* = 0.1126
Age (year)	35.2 ± 13.5	44.6 ± 13.9	36.5 ± 12.8	*P* = 0.007
TB culture**				
N/A (*n*, %)	0	0	0	
Negative (*n*, %)	0	38 (100%)	43 (100%)	
Positive (*n*, %)	26(100%)	0	0	
IGRA**				
N/A (*n*, %)	1 (3.8%)	0	0	
Negative (*n*, %)	1 (3.8%)	0	43 (100%)	
Positive (*n*, %)	24 (92.3%)	38 (100%)	0	

*PTB* pulmonary tuberculosis, *LTBI* latent tuberculosis infection, *HC* healthy control, *N/A* not available

^*^Statistical analyses for age data analyzed using the ordinary one-way ANOVA (3 groups): *P* = 0.007; unpaired *t* test (2 groups): PTB and LTBI *P* = 0.093; PTB and HC *P* = 0.7

Statistical analyses for sex data analyzed using the Chi-square test

^**^Before initiation of anti-tuberculous therapy

### *Expression of CD27-CD38* + *in PTB patients after treatment*

**Fig. 2:**
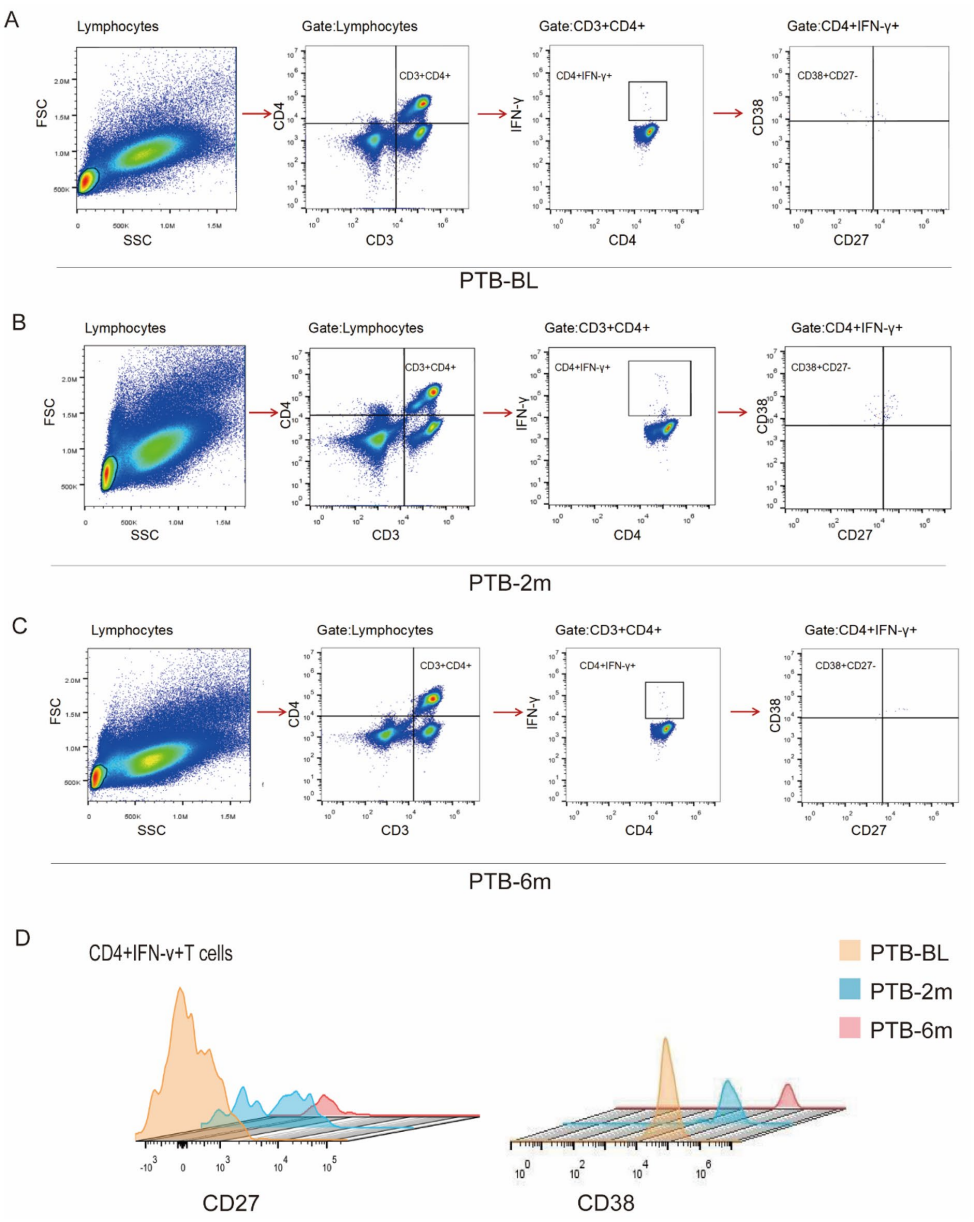
Characterization of CD27 and CD38 expression on TB-specific CD4^+^ T cells in whole blood of PTB patients at different treatment stages. Gating strategy to identify CD27 and CD38 expression on IFN-γ^+^CD4^+^ T cells in whole blood of PTB patients at stage of ATB-BL (**A**), stage of ATB-2 m (**B**) and stage of ATB-6 m (**C**). **D** The expression of CD27 and CD38 on TB-specific CD4^+^IFN-γ^+^ T cells in whole blood of ATB patients at different treatment stages. ATB-BL: ATB patients at baseline (untreated); *ATB-2 m* ATB patients after 2 months of treatment, *ATB- 6 m* ATB patients after 6 months of treatment

**Fig. 3:**
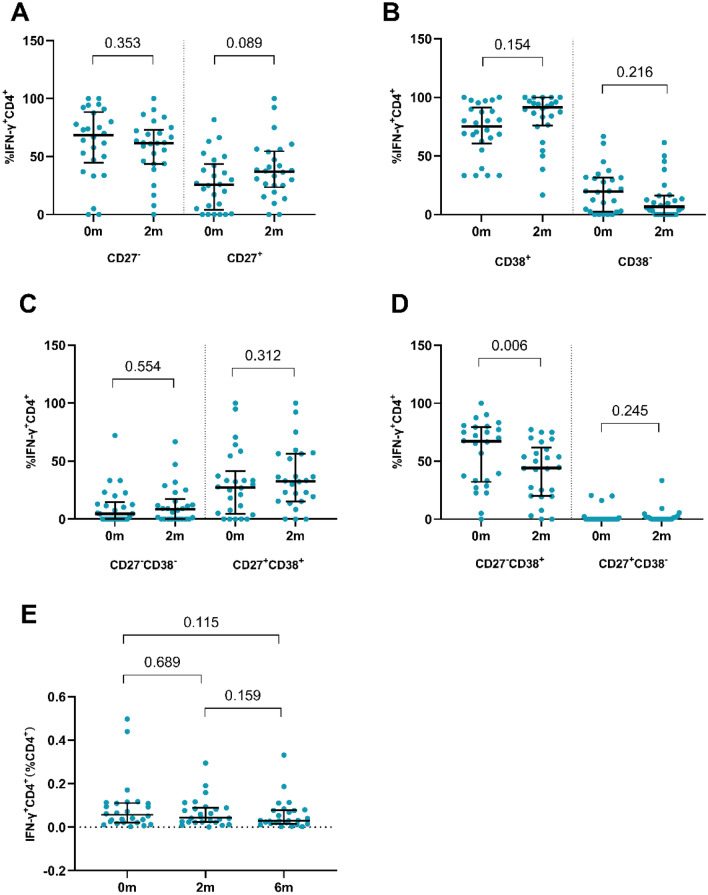
The frequency of phenotypic markers of CD27^−^ and CD27^+^ (**A**), CD38- and CD38^+^ (**B**), CD27^−^CD38^−^ and CD27^+^CD38^+^ (**C**) and CD27^−^CD38^+^ and CD27^+^CD38^−^ (**D**) on IFN-γ^+^CD4^+^ T cells in peripheral blood stimulated with ESAT-6/CFP-10 before and after 2 months of TB treatment. **E** The frequency of IFN-γ^+^CD4^+^ on CD4^+^ T cells in peripheral blood stimulated with ESAT-6/CFP-10 at baseline, 2 months and 6 months after TB treatment. Statistical analyses for paired data were performed using the Wilcoxon-signed rank paired test. Median values, interquartile range and *P*-values are indicated. *0 m* Before the TB treatment initiation, *2 m* 2 months after TB treatment initiation

The gating strategy of flow cytometry in determining CD27, CD38 and CD27CD38 is shown in Fig. 2. After being stimulated by the TB-specific antigen ESAT-6/CFP-10 for 16 h, CD27^−^CD38^+^IFN-γ^+^CD4^+^ cells are the main cell types (Fig. 2). The expression of CD27^−^, CD27^+^, CD38^−^, CD38^+^, CD27^−^CD38^−^, CD27^+^ CD38^+^, CD27^+^CD38^−^, CD27^−^CD38^+^ in IFN-γ^+^CD4^+^ cells before and after 2 months of treatment were further compared. As shown in Fig. 3A–D, only the expression of CD27^−^CD38^+^ was significantly decreased (*P* = 0.006).

**Fig. 4:**
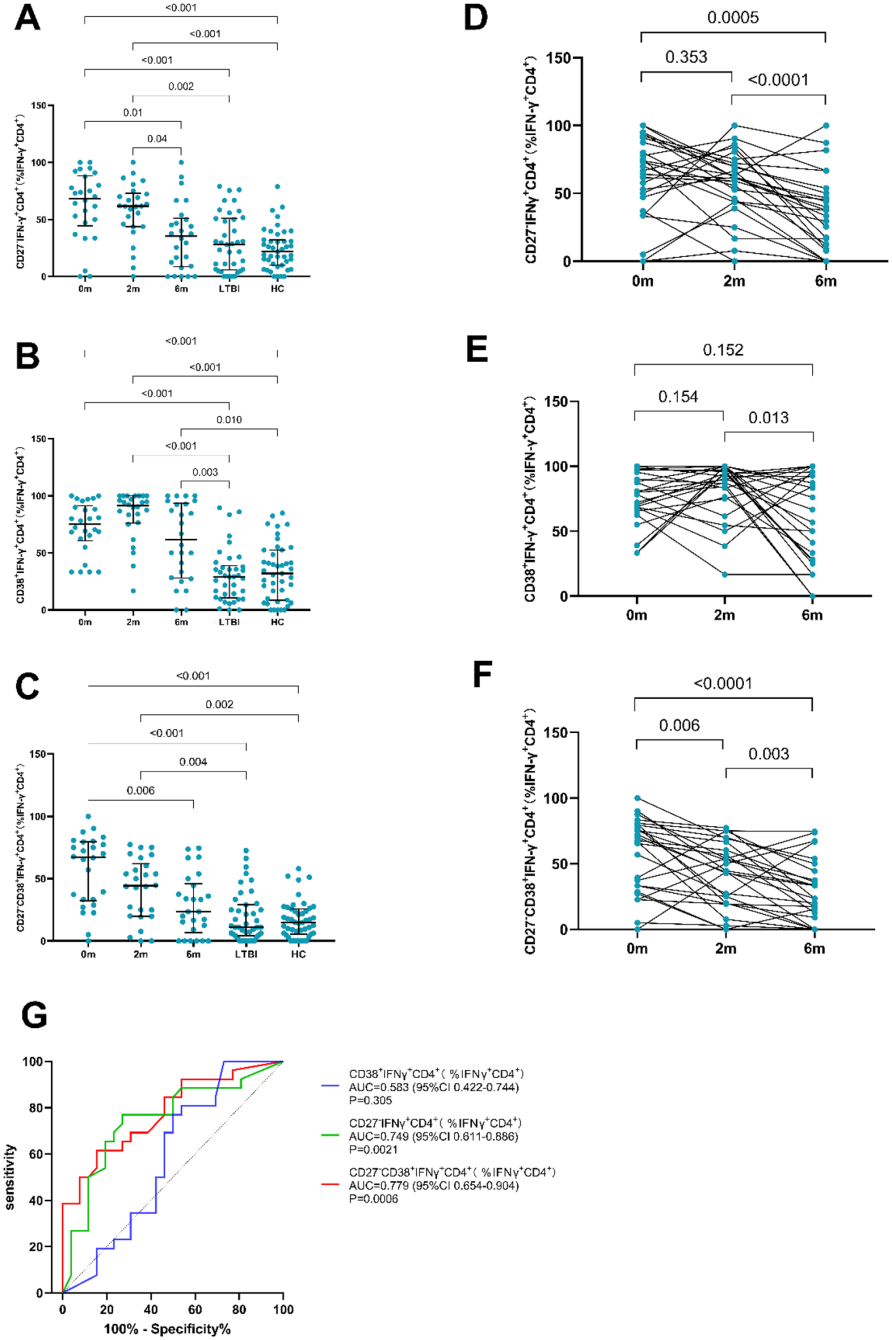
CD27^−^IFN-γ^+^CD4^+^ (**A**), CD38^+^IFN-γ^+^CD4^+^ (**B**) and CD27^−^CD38^+^IFN-γ^+^CD4^+^ (**C**) on IFN-γ^+^CD4^+^ T cells in peripheral blood stimulated with ESAT-6/CFP-10 in different groups were compared by the Kruskal–Wallis test. CD27^−^IFN-γ^+^CD4^+^ (**D**), CD38^+^IFN-γ^+^CD4^+^ (**E**) and CD27^−^CD38^+^IFN-γ^+^CD4^+^ (**F**) on IFN-γ^+^CD4^+^ T cells in peripheral blood stimulated with ESAT-6/CFP-10 in before and after 2 months or 6 months of TB treatment were compared pair-wise by the Wilcoxon-signed rank paired test. Median values, interquartile range, and *P*-values below 0.05 are indicated. Receiver operating characteristics (ROC) curves analysis of CD27^−^, CD38^+^, and CD27^−^CD38^+^ on IFN-γ^+^CD4^+^ T cells in peripheral blood stimulated with ESAT-6/CFP-10 comparing before and after 6 months of TB treatment (**G**). Area under the curve (AUC), 95%CI and P-values are indicated. *0 m* Before the TB treatment initiation, *2 m* 2 months after TB treatment initiation, *6 m* 6 months after TB treatment initiation, *LTBI* latent tuberculosis infection, *HC* healthy control

We further compared the IFN-γ (%CD4^+^) expression before the treatment, at 2 months and 6 months after treatment by paired comparison. As shown in Fig. 3E, there was no significant difference among the three time points (P values were all > 0.05), suggesting that the expression of IFN-γ in CD4^+^ T cells was not influenced by anti-TB treatment. The expressions of CD27^−^, CD38^+^ and CD27^−^CD38^+^ in PTB patients before and after 2- and 6-month treatment were compared with that in LTBI participants and healthy individuals (Fig. 4). The expressions of CD27^−^, CD38^+^ and CD27^−^CD38^+^ at baseline in PTB patients were significantly higher than that in LTBI participants and healthy individuals (*P* < 0.05). During the anti-TB treatment, the expressions of CD27^−^ and CD27^−^CD38^+^ were gradually decreased and was comparable to those in LTBI participants and healthy individuals after 6 months of anti-TB treatment (*P* > 0.05, Fig. 4A and C). However, the CD38^+^ expression was still significantly higher than that in LTBI participants and healthy individuals (*P* = 0.003 and 0.01, Fig. 4B) when the 6-month anti-TB treatment course was completed.

Further, we performed pair-wise comparison of the expressions of CD27^−^, CD38^+^ and CD27^−^CD38^+^ in PTB patients at different treatment stages. As shown in Fig. 4B, D and E, the expression difference of CD27^−^ was statistically significant between baseline and 6 m as well as between 2 and 6 m (*P* < 0.05). The expression difference of CD27^−^CD38^+^ between baseline and 2 m, 2 m and 6 m as well as baseline and 6 m was all statistically significant (*P* < 0.05). For the expression of CD38, statistical significance was only found between the 2 m and 6 m comparisons (*P* < 0.05).

### ***Diagnosis value of CD27***^***−***^***, CD38***^+^***and CD27***^***−***^***CD38***^+^***in evaluating the treatment efficacy***

The receiver operating curve (ROC) of CD27^−^, CD38^+^ and CD27^−^CD38^+^ before and after 6 months of anti-TB treatment was draw, and the area under the curve (AUC) was calculated. The AUC of CD27^−^IFN-γ^+^CD4^+^ (%IFN-γ^+^CD4^+^) (AUC = 0.749, *P* = 0.002), CD27^−^CD38^+^IFN-γ^+^CD4^+^ (%IFN-γ^+^CD4^+^) (AUC = 0.779, *P* = 0.0006) was relatively high (Fig. 4G), suggesting they were able to distinguish PTB patients before and after treatment. The AUC of CD27^−^CD38^+^IFN-γ^+^CD4^+^ (% IFN-γ^+^CD4^+^) was the highest, which was 0.779. Delong test indicated the difference between the AUC of CD27^−^IFN-γ^+^CD4^+^ (% IFN-γ^+^CD4^+^) and CD38^+^IFN-γ^+^CD4^+^ (% IFN-γ^+^CD4^+^) (*P* = 0.0439), as well as the AUC between CD27^−^CD38^+^IFN-γ^+^CD4^+^ (% IFN-γ^+^CD4^+^) and CD38^+^IFN-γ^+^CD4^+^ (%IFN-γ^+^CD4^+^) (*P* = 0.0123) were statistically significant. The cut-off values, sensitivity, and specificity of CD27^−^, CD38^+^ and CD27^−^CD38^+^ are shown in Table 2. The sensitivity of CD27^−^CD38^+^CD4^+^IFN-γ^+^CD4^+^ (%IFN-γ^+^CD4^+^) was 61.54% and the specificity was 84.62%, and the sensitivity of CD27^−^IFN-γ^+^CD4^+^ (% IFN-γ^+^CD4^+^) was 74.07% and the specificity was 76% (Table 2).

**Table 2:** Performance of different phenotypic markers

	Cutoff (%)	Se (%)	Sp (%)	FPR	FNR	PPV (%)	NPV (%)	+ LR	−LR	Youden
CD27^−^CD38^+^	55.41	61.54	84.62	15.38	38.46	80	68.75	4	0.455	0.4616
CD27^−^	46.9	76.92	73.08	26.92	23.08	74.07	76	2.857	0.316	0.50
CD38^+^	30.82	100	26.92	73.08	0	57.78	100	1.368	0.000	0.2692

*Se* sensitivity, *Sp* specificity, *FPR* false positive rate, *FNR* false-negative rate, *PPV* positive predictive value, *NPV* negative predictive value, ^*+*^*LR* positive likelihood ratio, ^*−*^*LR* negative likelihood ratio

### Correlation between phenotypic markers of IFN-γ^+^CD4^+^ cells and TB clinical indicators

**Fig. 5:**
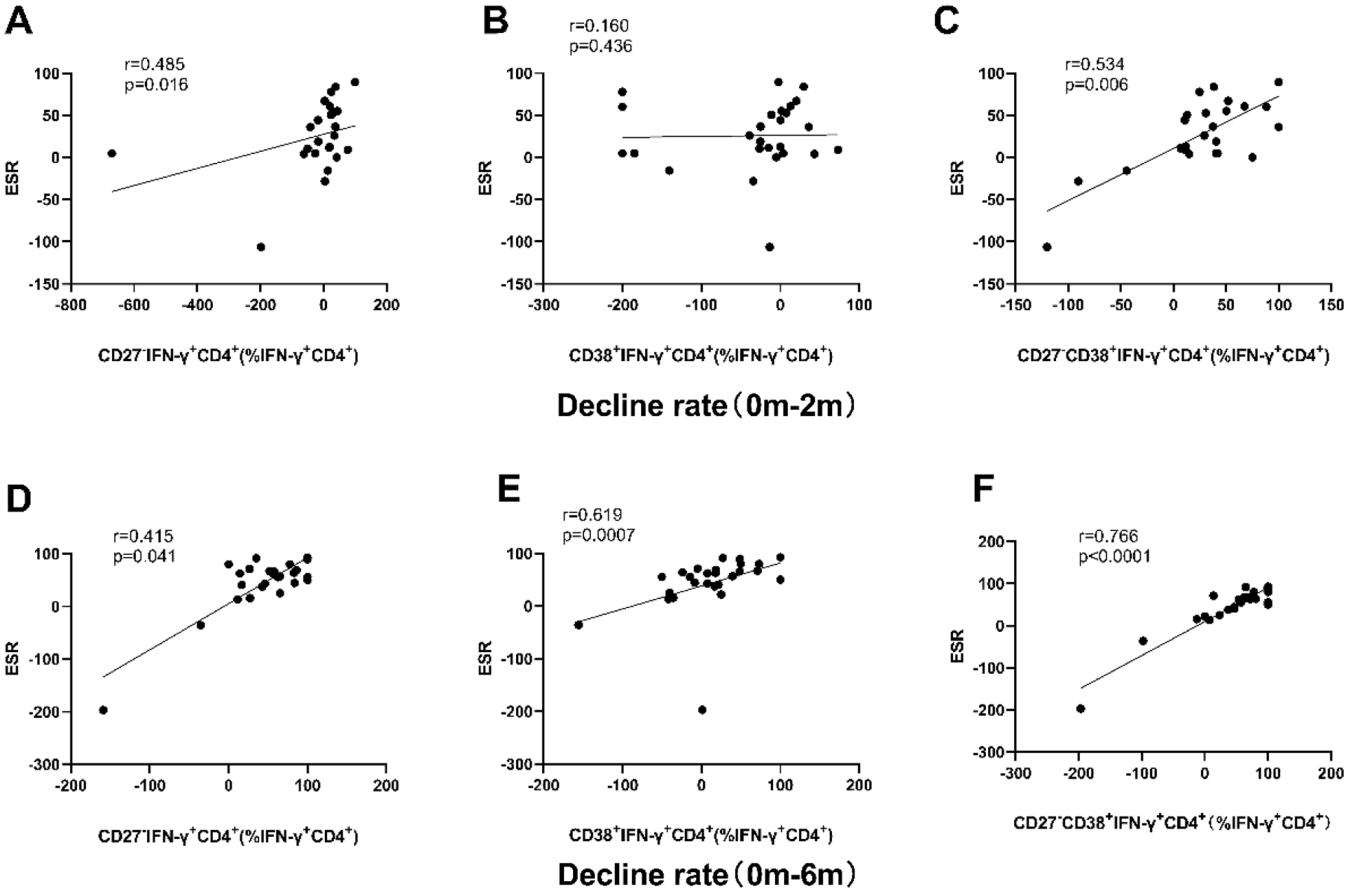
Correlation between the decline rate of phenotypic markers in IFN-γ^+^CD4^+^ cells in peripheral blood stimulated with ESAT 6/CFP-10 and that of ESR from before TB treatment to two or six months after TB treatment. Correlation of the decline rate between CD27^−^ and ESR (**A**), CD38^+^ and ESR (**B**), and CD27^−^CD38^+^ and ESR (**C**) from 0 to 2 m were analyzed; correlation of the decline rate between CD27^−^ and ESR (**D**), CD38^+^ and ESR (**E**), and CD27^−^CD38^+^ and ESR (**F**) from 0 to 6 m were analyzed. Statistical analyses were performed using the spearman test. r and P-values are indicated. *0 m* Before the TB treatment initiation, *2 m* two months after TB treatment initiation, *ESR* erythrocyte sedimentation rate

The correlation between the decline rate of CD27^−^, CD38^+^ and CD27^−^CD38^+^ in PTB patients before and after treatment and the decline rate of TB clinical indicators, including ESR, CRP and chest CT score was analyzed. As shown in Fig. 5, the decline rates (0 m–2 m) of CD27^−^ (*r* = 0.485, *P* = 0.016) and CD27^−^CD38^+^ (*r* = 0.534, *P* = 0.006) were significantly correlated with the decline rate of ESR. The decline rates (0 m-6 m) of CD27^−^ (*r* = 0.415, *P* = 0.041), CD38^+^ (*r* = 0.619, *P* = 0.0007), and CD27^−^CD38^+^ (*r* = 0.766, *P* < 0.0001) was significantly positively correlated with the decline rate of ESR, among which CD27^−^CD38^+^ had the highest correlation coefficient.

**Fig. 6:**
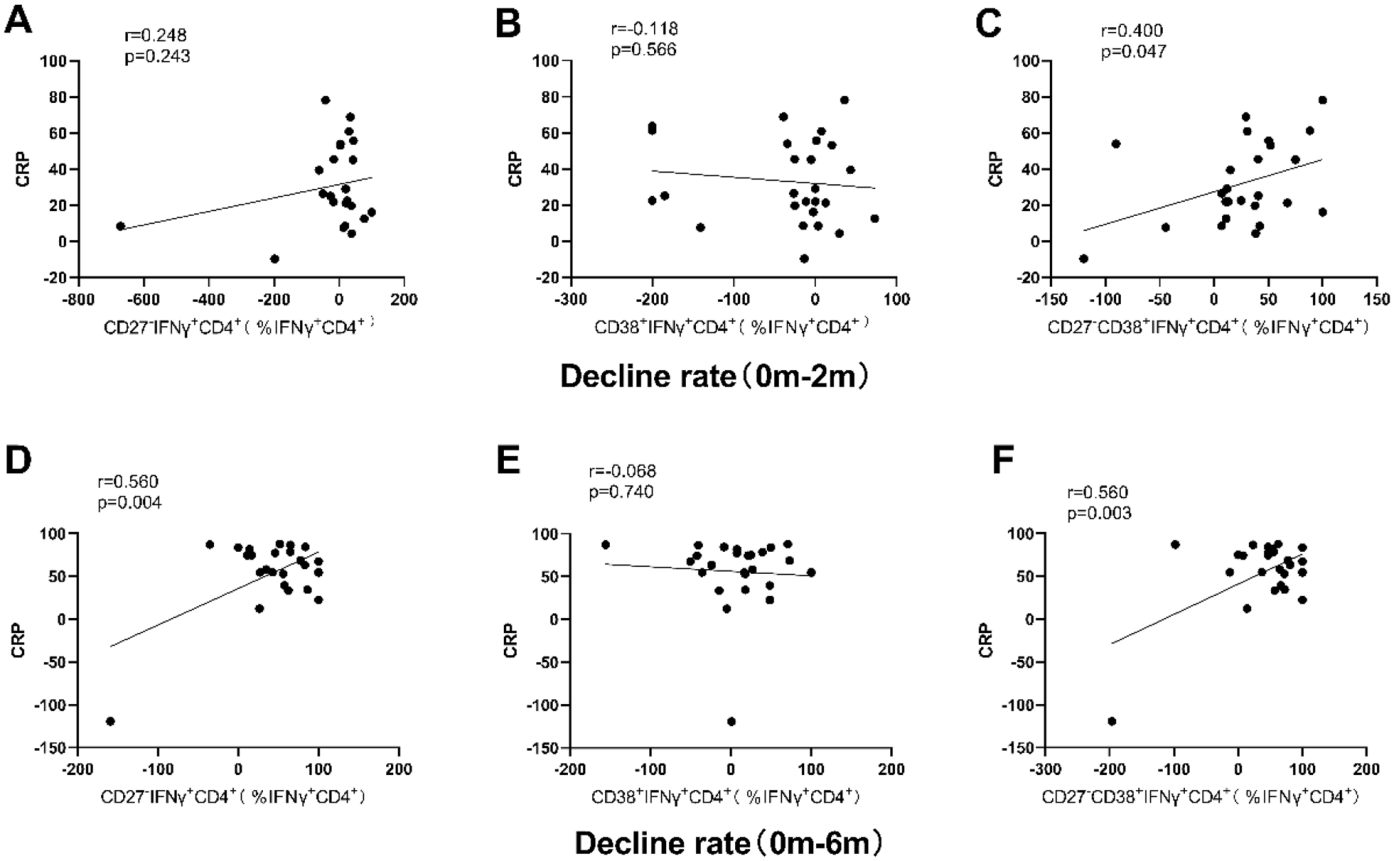
Correlation between the decline rate of phenotypic markers in IFN-γ^+^CD4^+^ cell in peripheral blood stimulated with ESAT 6/CFP-10 and that of CRP from before TB treatment to two or six months after TB treatment. Correlation of the decline rate between CD27^−^ and CRP (**A**), CD38^+^ and CRP (**B**) and CD27^−^CD38^+^ and CRP (**C**) from 0 to 2 m were analyzed; correlation of the decline rate between CD27^−^ and CRP (**D**), CD38^+^ and CRP (**E**), and CD27^−^CD38^+^ and CRP (**F**) from 0 to 6 m were analyzed. Statistical analyses were performed using the Spearman test. r and P-values are indicated. *0 m* Before the TB treatment initiation, *2 m* two months after TB treatment initiation, *CRP* C-reactive protein

As shown in Fig. 6, only the decline rate (0 m–2 m) of CD27^−^CD38^+^ (*r* = 0.400, *P* = 0.047) was significantly correlated with the decline rate of CRP. The decline rate (0 m–6 m) of CD27^−^ (*r* = 0.560, *P* = 0.004) and CD27^−^CD38^+^ (*r* = 0.560, *P* = 0.003) was significantly positively correlated with the decline rate of CRP.

**Fig. 7:**
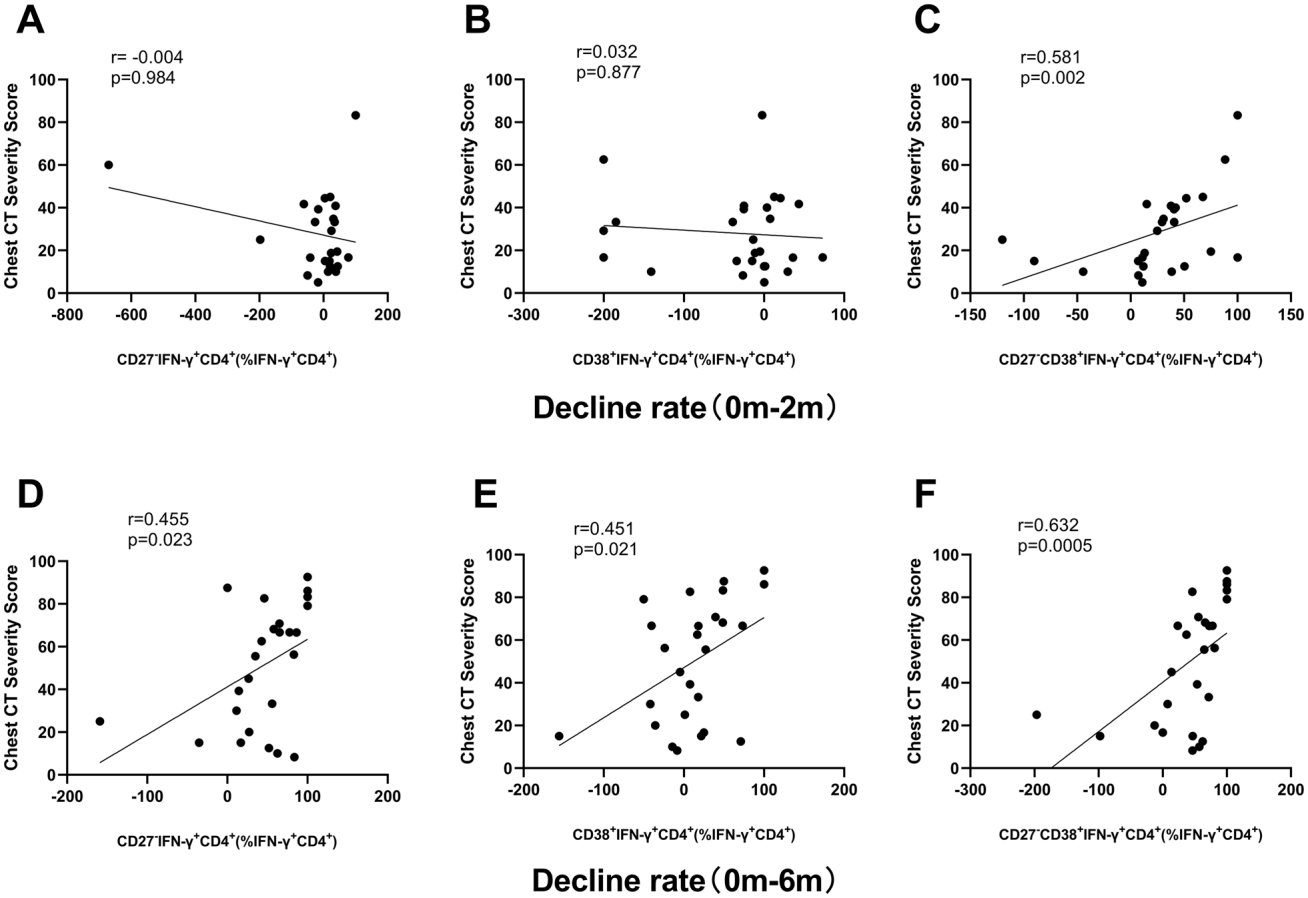
Correlation between the decline rate of phenotypic markers in IFN-γ^+^CD4^+^ cell in peripheral blood stimulated with ESAT 6/CFP-10 and that of chest CT severity score from before TB treatment to two or six months after TB treatment. Correlation of the decline rate between CD27^−^ and chest CT severity score (**A**), CD38^+^ and chest CT severity score (**B**), and CD27^−^CD38^+^ and chest CT severity score (**C**) from 0 to 2 m were analyzed; Correlation of the decline rate between CD27^−^ and chest CT severity score (**D**), CD38^+^ and chest CT severity score (**E**), and CD27^−^CD38^+^ and chest CT severity score (**F**) from 0 to 6 m were analyzed. Statistical analyses were performed using the spearman test. r and P-values are indicated. *0 m* Before the TB treatment initiation, *2 m* 2 months after TB treatment initiation

Only the decline rate (0 m-2 m) of CD27^−^CD38^+^ was significantly correlated with the decline rate of chest CT severity score (*r* = 0.581, *P* = 0.002). The correlation between the decline rate (0 m-6 m) of CD27- (*r* = 0.455, *P* = 0.023), CD38^+^ (*r* = 0.451, *P* = 0.021), CD27^−^CD38^+^ (*r* = 0.632,* P* = 0.0005) and the decline rate of chest CT severity score were statistically significant, among which, the correlation coefficient of CD27^−^CD38^+^ was the highest (Fig. 7).

## Discussion

At present, the main methods for evaluating the efficacy of anti-TB treatment are bacteriological test and chest radiograph. For bacterial-negative TB and some extrapulmonary TB, bacteriological test is useless. In addition, for some LTBIs that need to be treated with chemical drugs, radiograph is also unreliable. Thus, blood-based markers are proposed as an attractive alternative to diagnose, monitor and predict treatment response for the current sputum-based methods.

Since the cellular immune response mediated by T lymphocytes plays a key role in the process of TB infection and pathogenesis, immunological methods are considered to be used to evaluate the efficacy of TB treatment. IGRAs have high diagnostic value for TB infection and can identify TB-infected and non-infected people. Bugiani et al. studied the response of IGRA in TB patients after anti-TB treatment, and found that effective anti-TB therapy seems to restore T cell responses to MTB antigens, and the potential use of IGRA in monitoring TB treatment response is hampered by the presence of active mycobacterial replication at baseline [15]. Chiappini et al. also found that serial IGRA have limited use in children being treated with anti-TB therapy [16]. In this study, we compared the IFN-γ (%CD4 +) expression before the treatment, at 2 months and 6 months after treatment, and found there was no significant difference among these three time points. This result suggested that the expression of IFN-γ in CD4^+^ T cells was not influenced by anti-TB treatment, which was consistent with the previous studies [15, 16]. A systematic review also suggested that there was no uniform pattern of IGRA conversion and reversion at the end of treatment for active or latent TB, and in most studies, IGRA remained positive at the end of TB treatment [17]. Collectively, the variability among subjects suggests that IGRAs are unlikely to effectively monitor patients' anti-TB treatment in clinical practice.

In recent years, many studies have analyzed the expression changes of T cell phenotypic markers CD27 and CD38 during anti-TB treatment. Xu et al. found that the percentage of CD27^−^IFN-γ^+^CD4^+^ cells was significantly decreased after 3- and 6-month of anti-TB treatment [10]. Ahmed et al. [20] demonstrated that the frequencies of activation marker CD38^+^ were significantly decreased, while the frequencies of CD27- was largely unchanged until week 26 [18]. Vickers showed that after 2 months of anti-TB treatment, the proportion of CD27^+^CD38^+^CD4^+^ cells after ESAT-6/CFP-10 stimulation was significantly increased compared with the baseline [19]. After purified protein derivative stimulation, the proportion of CD8^+^CD27^−^IFN-γ^+^ and CD27^+^CD38^+^CD4^+^ T cells in subjects with slow treatment responders were significantly higher than those in subjects with fast treatment responders [19]. These studies suggested that CD27^−^ and CD38^+^ decrease is the main manifestations for effective anti-TB treatment. However, this study demonstrated that only the expression of CD27^−^CD38^+^ was significantly decreased after 2 months of anti-TB treatment compared with baseline.

Subsequently, we compared the expression of CD27^−^, CD38^+^ and CD27^−^CD38^+^ in IFN-γ^+^CD4^+^ cells and found that at the completion of the 6-month treatment course, the expression of CD27^−^ and CD27^−^CD38^+^ in the PTB group was comparable with those in LTBI and healthy controls, while the expression of CD38^+^ was not significantly influenced by the anti-TB treatment. These results suggested that the expression of CD27^−^ and CD27^−^CD38^+^ in IFN-γ^+^CD4^+^ cells might be markers for monitoring anti-TB treatment. The ROC curves of each index before the initiation of anti-TB treatment and after 6 months of anti-TB treatment were drawn, and the AUCs indicated the expression of CD27^−^ and CD27^−^CD38^+^ in IFN-γ^+^CD4^+^ T cells might be potential markers to distinguish patients with active TB before and after treatment. The AUC of CD27^−^CD38^+^IFN-γ^+^CD4^+^ (% IFN-γ^+^CD4^+^) was the highest at 0.779 with a sensitivity of 61.54% and a specificity of 84.62%.

The speed of ESR can help to observe the changes of the disease, including rheumatism and TB. In some studies [20, 21], the expression changes of CD27^−^CD38^+^ in IFN-γ^+^CD4^+^ cells between 2 months after treatment and baseline was positively correlated with ESR changes. In our study, the expression changes of CD27^−^, CD38^+^ and CD27^−^CD38^+^ were positively correlated with ESR at 6 months after treatment, and CD27^−^CD38^+^ was the most closely correlated one. CRP is a non-specific marker of inflammation and tissue damage, and CRP is significantly increased when the lower respiratory tract is infected with bacteria [22, 23]. The results of our study showed that only the decline rate (0 m–6 m) of CD27^−^ and CD27^−^CD38^+^ was significantly positively correlated with the decline rate of CRP. These results suggested that the expression of CD27^−^CD38^+^ in IFN-γ^+^CD4^+^ cells can well reflect the degree of infection and its changes. In addition, the expression changes of CD27^−^, CD38^+^, CD27^−^CD38^+^ at 6 months of treatment were positively correlated with chest CT severity scores, and among which, CD27^−^CD38^+^ had the closest correlation.

This study proposed a new method to predict the efficacy of anti-TB treatment. However, there are still some limitations in this study. The number of cases for efficacy evaluation is relatively small and we did not split the data into training set and test set due to the limited number of patients. Besides, quantitative detection of TB was not performed, which lacks an important criterion for the change of the condition before and after anti-TB treatment. Therefore, future research on large population is still warrant to validate our study.

In conclusion, the expressions of CD27^−^, CD38^+^ and CD27^−^CD38^+^ in IFN-γ^+^ CD4 + cells are decreased after anti-TB treatment, but the expression of CD27^−^CD38^+^ can relatively more accurately to reflect the efficacy of anti-TB treatment. The change of CD27^−^CD38^+^ in IFN-γ^+^CD4^+^ cells before and after TB treatment is expected to become a potential new marker for evaluating the efficacy of anti-TB treatment.

## Data Availability

The datasets used and/or analyzed during the current study are available from the corresponding author on reasonable request.
